# The MeLiM Minipig: An Original Spontaneous Model to Explore Cutaneous Melanoma Genetic Basis

**DOI:** 10.3389/fgene.2017.00146

**Published:** 2017-10-13

**Authors:** Emmanuelle Bourneuf

**Affiliations:** ^1^LREG, CEA, Université Paris-Saclay, Jouy-en-Josas, France; ^2^GABI, INRA, AgroParisTech, Université Paris-Saclay, Jouy-en-Josas, France

**Keywords:** porcine model, predisposition genes, cutaneous melanoma, QTL, endogenous retrovirus

## Abstract

Melanoma is the deadliest skin cancer and is a major public health concern with a growing incidence worldwide. As for other complex diseases, animal models are needed in order to better understand the mechanisms leading to pathology, identify potential biomarkers to be used in the clinics, and eventually molecular targets for therapeutic solutions. Cutaneous melanoma, arising from skin melanocytes, is mainly caused by environmental factors such as UV radiation; however a significant genetic component participates in the etiology of the disease. The pig is a recognized model for spontaneous development of melanoma with features similar to the human ones, followed by a complete regression and a vitiligo-like depigmentation. Three different pig models (MeLiM, Sinclair, and MMS-Troll) have been maintained through the last decades, and different genetic studies have evidenced a complex inheritance of the disease. As in humans, pigmentation seems to play a prominent role, notably through MC1R and MITF signaling. Conversely, cell cycle genes as *CDKN2A* and *CDK4* have been excluded as predisposing for melanoma in MeLiM. So far, only sparse studies have focused on somatic changes occurring during oncogenesis, and have revealed major cytological changes and a potential dysfunction of the telomere maintenance system. Finally, the spontaneous tumor progression and regression occurring in these models could shed light on the interplay between endogenous retroviruses, melanomagenesis, and adaptive immune response.

## State of the Art of Melanoma Genetics

Melanoma is a tumor arising from melanocytes, highly differentiated cells notably found in the skin of Vertebrates. Melanocytes are derived from the neural crest of the embryo, from which melanoblasts migrate to colonize the skin of the entire body, the eye, as well as other structures. Later, melanoblasts differentiate into functional melanocytes, which main role is pigment production (Cichorek et al., 2013). Melanins (black eumelanin and red pheomelanin) are synthesized mainly to prevent DNA damage due to UV radiation (Miyamura et al., 2007). However, sun exposure, as well as other environmental or intrinsic factors, can lead to malignant transformation of melanocytes, thus turning into melanoma. Several clinical types of melanoma prevail: cutaneous melanoma, uveal melanoma developing in the eye, acral melanoma observed on palms of hands, soles of feet and nails, and mucosal melanoma, located in the nose, mouth, vagina, urinary tract, and rectum for example. Other less frequent subtypes are described, and all these different forms of melanoma have different causalities, molecular mechanisms and outcomes (Schadendorf et al., 2015). This review will focus only on cutaneous melanoma, which also displays heterogeneous histological features. For example, the two main subtypes are Nodular Melanoma [or Vertical Growth Phase melanoma (VGP)], and Superficial Spreading Melanoma [or Radial Growth Phase Melanoma (RGP)]. Other melanocytic lesions can be observed (nevi, lentigo, atypical melanocytic proliferations…) but most remain benign and do not lead to malignant transformation.

The incidence of cutaneous malignant melanoma has been increasing for decades, reaching in 2011 a rate of around 30 per 100,000 per year in United States whites, Swedish and Norwegian populations, a rate of 19.8 per 100,000 in United Kingdom, and up to 51 per 100,000 in Australian and New Zealand populations (Whiteman et al., 2016). Mortality associated to melanoma has also been increasing in all six populations, although at a slower pace. The highest incidence is observed in fair-skinned populations; however dark-skinned individuals also present cutaneous melanoma, usually diagnosed at a later stage with a greater Breslow thickness, and associated with a poorer outcome (Hore et al., 2010). Also, non-caucasian populations have a higher incidence of acral lentiginous melanoma, thus independent of sun exposition (Stubblefield and Kelly, 2014). The only efficient treatment for cutaneous melanoma is *in situ* resection of the primary tumor, when no cells have spread away in the body. Once a metastatic process has started, most of classical therapies used in oncology do not improve survival (Schadendorf et al., 2015). Nevertheless, recent developments in immunotherapy have shown exciting results, with the use of antibodies targeting immune checkpoints such as CTLA-4 and PD-1 (Boutros et al., 2016). Melanoma is among the most immunogenic cancers, with an important mutational load, probably explaining a high rate of partial tumor regression. Nevertheless, to circumvent this potential antitumor response, melanoma cells profit from immune checkpoints such as CTLA-4 and PD-1 pathways. These surface molecules, expressed notably by T lymphocytes, are negative regulators of immune function, and prevent an over-activation of immune response in physiological conditions. Thus, immune checkpoints inhibition stimulates the host immune response, via diverse mechanisms: CTLA-4 pathway blockade allows for proliferation and activation of more T-cells, while PD-1 blockade restores of the antitumor activity of T cells. These treatments have shown unprecedented responses and antitumor activity in advanced melanoma patients, and extensive clinical trials are currently underway (Boutros et al., 2016).

Cutaneous melanoma is mainly due to exposure to UV radiation, and strong prevention campaigns around a limited use of sunbeds and extensive protection with sunscreens has proven efficient in the last years (Lo and Fisher, 2014). However, genetic susceptibility has been shown to be another factor promoting melanoma development (Aoude et al., 2015b), in around 10% of patients (Florell et al., 2005). Thus, sporadic melanomas can be distinguished from familial melanoma cases, for which genetic studies can be considered. Other factors of importance are for example a high number of nevi on the body, or the patient’s skin phototype, with an increased risk of melanoma if carrying >50 moles or in fair-skinned people (MacKie et al., 2009).

In human, early linkage analyses performed in large families have led to the identification of two high-risk genes, namely *CDKN2A* and *CDK4*, both cell cycle regulators. *CDKN2A* is the most important gene described so far, and around 20% of familial cases are due to deleterious mutations in this gene (Hussussian et al., 1994; Kamb et al., 1994; Potrony et al., 2015 for review). Loss-of-function mutations thus discard the inhibitory role of the two *CDKN2A* products, namely p16^INK4A^ and p14^ARF^ on cell cycle progression, leading to enhanced proliferation. The *CDK4* R24C activating mutation is also highly penetrant, although it concerns only a few families worldwide (Zuo et al., 1996; Soufir et al., 1998): this non-synonymous substitution changes an amino acid essential for binding of CDK4 to p16^INK4A^, leading to an increased proliferation. Early studies have also designated *MC1R* as a low-risk gene (Valverde et al., 1996; Palmer et al., 2000). *MC1R* codes for a G-protein-coupled receptor, which physiological ligand is α-MSH. A binding to MC1R leads to an increase in cAMP production, and therefore an activation of subsequent cascades in the cell. In melanocytes, *MC1R* expression controls the balance between pheomelanin and eumelanin production and thus regulates the pigmentation of the individual (Dessinioti et al., 2011). For example, a series of polymorphisms, named RHC variants for Red Hair Color variants, are carried by individuals with light skin, blue eyes and red hair, poor tanning ability and sensitivity to sunburn (Flanagan et al., 2000). These patients also display a higher risk of melanoma, initially related only to the role of *MC1R* in pigmentation. Further studies have shown a much more complex picture of *MC1R* effects in the melanocyte: in addition to the UV-protective effect of melanin, *MC1R* can influence melanoma beyond pigmentation, through the positive effect of cAMP on repair of UV-induced damage (Hauser et al., 2006; Kadekaro et al., 2010). In two recent studies focusing on somatic mutations in melanoma, the authors reported a more important mutational burden in tumors of patients carriers of 1 or 2 RHC alleles compared to non-RHC patients (Robles-Espinoza et al., 2016; Johansson et al., 2017). Also, this higher mutation rate is observed for all mutation classes, and not only the UV damage-associated C>T transitions, indicating other potential mutational processes linked to *MC1R* germline variation.

Since 2011, the *MITF* gene (Microphtalmia-Associated Transcription Factor) is considered as a medium-risk gene, since two studies involving linkage analysis followed by sequencing, showed the functional impact of the E318K rare mutation on melanoma and renal cell carcinoma risks (Bertolotto et al., 2011; Yokoyama et al., 2011). The mutation impairs the SUMOylation of MITF proteins, leading to a differential transcriptional activity of the target genes. More recently, Bonet et al. (2017) demonstrated that human melanocytes carrying the *MITF* E318K mutation could no longer undergo BRAF^V 600E^-induced senescence, thus promoting melanoma development. Horn et al. (2013), mutations in *TERT* promoter were shown to modify melanoma risk in familial melanoma. In addition, TERT promoter is frequently mutated in sporadic melanomas as well as other tumor types (Aoude et al., 2015b).

With the advent of high-throughput technologies such as SNP genotyping and sequencing, numerous Genome-Wide Association Studies have been performed in large case-control designs, for melanoma occurrence *per se*, as well as connected phenotypes such as number of nevi and pigmentation (reviewed in Law et al., 2012). These approaches have pointed at several genes which role in melanoma development remains to be elucidated. However, a number of genes associated with melanoma are involved in pigmentation (*ASIP, OCA2, TYR, TYRP1*, and *MTAP*), or in cell cycle regulation (*CCND1*, *CDKAL1*). Other function classes include immune response (*HLA*, *IRF4*) and metabolism (*FTO*, *VDR*). Most of these results have been confirmed in a recent international meta-analysis (Law et al., 2015).

Finally, successful candidate genes approaches led to the identification of other high risk genes more recently, like for example *BAP1* for which germinal mutations are found in a wide spectrum of neoplasms (Wiesner et al., 2011; Carbone et al., 2013). Since the discovery of predisposing variants in *TERT* promoter, research on melanoma genetics has focused on germline mutations in genes coding for components of the telomere maintenance complex. Mutations in *POT1* (Robles-Espinoza et al., 2014; Shi et al., 2014), and in *ACD* and *TERF2IP* (Aoude et al., 2015a) have been associated with melanoma increased risk in around 1% of predisposed families (Potrony et al., 2015). As a consequence, a renewed interest has grown for telomere maintenance in tumors as a potential therapeutic target, despite previous pitfalls (Zanetti, 2017).

Overall, different approaches allowed the identification of low to high risk genes, and of common variants showing a very limited effect on disease risk when taken alone. One of the major difficulties in GWAS of complex traits is genetic heterogeneity of the cases, which requires the use of very large cohorts and careful clinical classification of the patients. A possible way to bypass this critical issue is the use of animal models to better understand the complex genetic and molecular mechanisms leading to melanomagenesis.

## Animal Models of Melanoma

The most frequently used model for melanoma is the mouse. Spontaneous melanoma is very rare in mouse, but the model is notably used as a support for patient-derived xenografts (PDXs) in immunocompromised animals. PDX mice properly model the human disease and can guide personalized therapy decisions (Hartsough and Aplin, 2016). Another essential application of mouse as a model is the relatively easy manipulation of its genome. Therefore, a large set of GEM (genetically engineered mice) models were developed and used for a fine dissection of molecular pathways governing melanocyte transformation and melanoma progression. For example, Mann et al. (2015) used transposon mutagenesis in a BRAF^V 600E^ mouse model to determine a set of genes cooperating with the BRAF mutation to drive melanoma progression. Those fine studies performed in GEM mice can give crucial information about pivotal pathways and potential therapeutic targets for human melanoma.

A major limitation to the use of mouse as a model is that melanoma incidence is often very low and appears at late onset (van der Weyden et al., 2015). In addition, murine melanocytes reside in the dermis, and not at the basal layer of the epidermis. This latter aspect should be taken into account since a large part of melanoma biology (and cancer in general) is modulated by surrounding stromal cells and immediate environment. To circumvent this anatomical issue, another interesting mouse model has been developed, by overexpressing the HGF/SF (hepatocyte growth factor/scatter factor) under the metallothionein promoter (Takayama et al., 1997). In the HGF/SF mouse model, melanocytes colonize not only the dermis, but also the epidermis. In addition, HGF/SF mice harbor different histological types of lesions, including sporadic melanomas arising following UV exposition. Nevertheless, despite a very convenient handling, and the undeniable advantage of genetic engineering to modulate single genes function, mouse models may not recapitulate all the features of a complex disease. The lack of translatability to human of mouse results obtained on inflammatory diseases illustrates this aspect (Seok et al., 2013). Naturally occurring models may better reflect complexity and might be closer to reality (**Table 1**).

**Table 1:** Examples of animal models of melanoma, and some of their advantages/disadvantages to explore melanomagenesis in human.

Species	Advantages	Disadvantages
Mouse	Genetic manipulation possible	Late onset
	Different genetic backgrounds available	Low incidence
	Easy breeding and handling	No spontaneous melanoma, genetic modifications needed
	Vast genetic and genomic resources	Melanocytes in dermis
	Many examples of molecular pathways dissection	
Pig	Cutaneous melanoma	Major susceptibility genes identified in human are not predisposing in pigs
	Early onset of multiple tumors	Early onset and UV-independent, thus not reflecting a large part of human cases occurring in the elderly, on sun-damaged skin
	No environmental effect	Cell biology tools are limited (antibodies for example)
	Same inheritance as humans	
	Common histological and clinical features with human melanoma, including metastatic invasion	
	Spontaneous and complete regression	
	Melanocytes on the basal layer of the epidermis	
Dog	Several possible clinical types (mucosal, cutaneous, acral, uveal)	Often benign (except melanomas from the oral cavity)
	Veterinary records	Cell biology tools are limited (antibodies for example)
	Anti-cancer treatments and clinical trials	Genetic basis remains poorly described
	Shared environment with human	
	Somatic mutations similar to human ones	
	Breed genetic structure should facilitate association analysis	
Horse	Presence of nevi and melanomas	Correspond to rare melanomas in human
	Dermal melanomas can eventually metastasize	Late metastatic evolution in gray horses
	The genetic basis of melanoma development in gray horses is partly known	
	Activation of ERK pathway, as seen in human	


Among non-rodent models for cutaneous melanoma, zebrafish is a very attractive model to decipher precise mechanisms (Michailidou et al., 2009), perform *in vivo* imaging (Heilmann et al., 2015) and drug screening (Xie et al., 2015). However, as in rodents, melanoma development is induced by genetic manipulation of oncogenes or ENU-induced mutagenesis, which is a major limitation to study genetic predisposition. Naturally occurring models include some horse breeds, for which aging is associated with graying of the coat color, mucosal melanomas and depigmentation. Melanoma development has been attributed to a mutation in the intron 6 of *STX17* (Rosengren Pielberg et al., 2008), leading to an activation of the ERK pathway (Jiang et al., 2014). Dogs show a quite high incidence of melanoma, and different breeds can serve as models for multiple melanoma subtypes. For example, cutaneous melanoma is found frequently in Beauce shepherds, while poodles are more prone to developing oral tumors (Gillard et al., 2014). Moreover, the structure of dog breeds has been shown as a very efficient tool for trait mapping, even for complex diseases (Rimbault and Ostrander, 2012). These extraordinary features also make the dog an exciting model for melanoma predisposition.

## Swine Models of Melanoma

Pigs and large animals in general are recognized as compelling models for human diseases. This wonderful potential has had a limited impact because of the poor availability of genomic resources for domestic species. However, recent technological breakthroughs now circumvent these pitfalls. For example, swine models of mutations found in human can now be produced to decipher the mutation effect *in vivo*. Thus, the very recent development of “oncopigs” is of major importance (Schook et al., 2015), but remains complementary to spontaneous models to reflect and model natural complexity.

One of the major advantages of swine models is the tightly controlled breeding process, so that the genetic determinism can be studied independently from potentially interacting environmental factors. Thus, a spontaneous porcine model of melanoma, bred totally indoors, can help deciphering melanoma genetics, without any influence of the UV-dependent mechanisms. Also, pig breeds harbor a limited genetic heterogeneity, mimicking to some extent a complex susceptibility background, but still allowing genetic studies with reasonable number of samples.

The pig has been used for skin physiological studies for decades, given its properties comparable to human skin (Vodicka et al., 2005). One of the most interesting point using pig as a model for a cutaneous melanoma is the location of the melanocytes, sitting on the basal layer of the epidermis, as in humans, and contrary to rodents where they are found in the dermis. Thus, swine skin is expected to better reflect the microenvironment of the healthy and transformed cells. This fundamental aspect is illustrated for example by a recent study showing that melanoma vertical invasion is governed by contact with keratinocytes in human (Golan et al., 2015). Another advantage of the swine model is the early onset of melanoma and high incidence in some specific breeds. As a consequence, clinical observations and sampling can be performed in the first weeks of the animals and do not require producing a large number of animals and waiting for tumor appearance. Yet, one should keep in mind that early onset melanoma is only rarely observed in human, and generally originates from a giant congenital nevus (Kinsler et al., 2017). Other pediatric melanomas appear rarely before puberty, and share features with adult melanomas developing on an intermittently sun-exposed skin (Lu et al., 2015).

Pigs bearing cutaneous melanoma have been described as early as in the 30s (Nordby, 1930). Commercial breeds also show a low incidence of melanoma. For example, a few cases were described in the progeny of a cross between a Duroc male and a Slovak White sow (Levkut et al., 1995), in the Hampshire breed (Empringham and Wilkins, 1979), or in slaughterhouses, without any mention on the breed (Bundza and Feltmate, 1990; Vidal et al., 2015). Frequent cases have been documented in Duroc swine, and in Duroc X Iberian cross (Thirloway et al., 1977; Hordinsky et al., 1985; Mishima et al., 1989; Perez et al., 2002). Interestingly, there is no mention in the literature of any melanoma lesions in Asian breeds.

In addition to these animals, three models have been selectively bred for cutaneous melanoma studies (**Table 2**). Their origins go back to two different breeds, Hormel and Hanford (Köhn, 2011). The first line is the Munich miniature swine (MMS) Troll, maintained and studied at the Institute of Veterinary Pathology, University of Munich (Germany). The animals are derived from Hanford and Columbian miniature swine, and have been selectively bred since 1986 (Müller et al., 2001). The Sinclair pig originates from the Hormel swine, and was first described in Millikan et al. (1974), when authors mentioned a melanoma incidence of 21% in the herd back in the 1960s. Since then, Sinclair melanoma was studied in different laboratories and is maintained now in the Sinclair Research Center in the United States http://www.sinclairresearch.com/. Finally, the Melanoblastoma-bearing Libechov Minipig (MeLiM) model was originally maintained in the Libechov Institute in Czech Republic, and further distributed to a French unit belonging to INRA and CEA (Geffrotin et al., 2000). Since then, only a few animals from the Czech herd have been imported to France, but animals still remain comparable. Horak et al. (1999) described the appearance of tumors in these animals: pigs from Hormel origin were crossed with Göttingen minipigs, with a white with black spots coat color, and later with four additional breeds (Canadian Landrace, Cornwall, Large White, and Vietnamese). The objective was to increase genetic variation in a herd designed for blood group variability studies, while maintaining a miniature phenotype. Cutaneous melanoma appeared in the herd in the 80s and was further selected for, so that the melanoma incidence reached more than 50% after some years. The fact that at least two of the three models (Sinclair and MeLiM) come from the Hormel swine farm would indicate shared genetic variants, potentially including the melanoma predisposing variants. Studying both models in parallel would therefore reinforce findings.

**Table 2:** Pathological and genetic features of the three main swine breeds bearing cutaneous malignant melanomas.

		MeLiM	Sinclair	MMS-Troll
Breed features	Origin	Hormel	Hormel	Hanford
	Coat color	Red or black		
Melanoma traits	Age of onset	At birth or in the first weeks		
	Clinics	Single or multiple lesions, clinical ulceration, local or distant metastasis		
	Histology	SSM and NM subtypes, presence of flat benign lesions, Clark’s level I-V, histological ulceration, skin invasion to dermis or subcutaneous adipose tissue, heavily pigmented melanoma cells and presence of pigment-laden macrophages		
	Regression	Spontaneous and total		
	Depigmentation	Partial or total, affecting hair, skin, and eye		
	Mode of inheritance	2–3 loci, or complex autosomal dominant with incomplete penetrance	One major unmapped locus + SLA “B haplotype”	For nevi: one major gene and polygenic background
				For melanoma: two to three recessive genes
				No influence of SLA
	Reference	Geffrotin et al., 2004; Hruban et al., 2004; Vincent-Naulleau et al., 2004; Du et al., 2007	Millikan et al., 1974; Hook et al., 1979; Tissot et al., 1987; Green et al., 1992; Misfeldt and Grimm, 1994; Gomez-Raya et al., 2007; Ho et al., 2010	Müller et al., 2001; Dieckhoff et al., 2007


In the three breeds, as well as sporadic cases above mentioned, melanoma exclusively occurs on colored animals (solid red or black coats). This observation is explained by the absence of melanocytes in the skin of white pigs, due to a complex mutation of the *KIT* gene (Johansson Moller et al., 1996). This gene codes for a tyrosine kinase receptor expressed at the cell surface and regulating several intracellular processes. KIT is notably present at the surface of melanoblasts and regulates the migration of cells in the embryo. Deleterious mutations in *KIT* thus impair melanoblasts migration, leading to a white coat color. Therefore, melanoma predisposing variants could not have an effect on a white animal that does not possess the cell of origin of the tumor.

In all animals, tumors appear early in life, and even before birth. In Sinclair, Beattie et al. (1988) have shown the presence of melanocytic hyperplasia as early as the 11th week of gestation. Tumors are exclusively cutaneous and no uveal or mucosal melanoma has been reported so far in pigs. There is no predilection for cutaneous tumor location on the body, reflecting the absence of environmental influence. In human, predictably, cutaneous melanomas are more frequently observed in sun-exposed areas of the body, although non-CSD (chronically sun-damaged) melanomas are not negligible (Shain and Bastian, 2016). Also, no sex difference has been observed in transmission of the disease in the three swine models.

The overall clinical presentation also shares features between breeds. Indeed, animals can carry multiple lesions, some being flat and benign, others showing obvious malignancy signs and eventually leading to metastasis (Millikan et al., 1974; Hook et al., 1979; Horak et al., 1999). Vincent-Naulleau et al. (2004), realized a more extensive clinical and histological description of the melanocytic lesions found in the MeLiM pig, along with a comparison with human classification. Three types of lesions are observed, from benign to highly invasive. The benign flat melanocytic lesions show no metastatic invasion, and are histologically similar to atypical melanocytic proliferations for the vast majority of them. A second histological subtype consists of raised and pigmented lesion but without malignant evolution (no ulceration, slow growth, no metastasis). Finally, heavily pigmented tumors with a rapid growth correspond to invasive melanoma. Some are exophytic and often exhibit ulceration, and eventually lymph nodes and visceral metastasis. Histologically, they correspond to SSM or NM, with a larger proportion of NM compared to human.

Importantly, the different models share the fascinating phenomenon of tumor regression. The regression process has been described in details, both clinically an histologically in MeLiM (Vincent-Naulleau et al., 2004). This corresponds to a spontaneous and complete disappearance of tumors and metastasis, without any treatment. Regression commonly occurs after a few weeks of age, between 2 and 4 months after birth. While the immune system intervention is indisputable, transcriptomic analyses performed in a MeLiM time-course experiment has shown a potential cell cycle arrest of the melanoma cells, occurring before the infiltration of the tumor by lymphocytes (Rambow et al., 2008a,b). The possible involvement of immune checkpoints in swine tumor regression has not been established yet.

Along with this fascinating process, a partial or total depigmentation of skin, hair and iris occurs, starts around the regressing lesions, and eventually propagates to the whole body for the totally depigmented individuals. Some pigs remain “spotted,” while others become totally white with blue iris. Misfeldt and Grimm also mention depigmentation in black Sinclair (Misfeldt and Grimm, 1994) and hypothesize that it could reflect an immune response toward melanocytes and melanoma. The nodal and visceral metastases also regress, giving way to fibrotic tissue. Overall, only a 4% mortality rate is observed, likely due to metastatic complications appearing before the regression onset (Vincent-Naulleau et al., 2004).

## Melanoma Inheritance in Pigs

Several studies have been conducted in the three models, with different approaches, and leading to different conclusions. In the MMS Troll, a first model is proposed for the inheritance of flat benign lesions (defined as nevi in this breed), i.e., the influence of one major gene on a polygenic background. For melanoma tumors however, two to three recessive genes may be involved (Müller et al., 2001). In Sinclair, several publications (Tissot et al., 1987; Blangero et al., 1996) describe a model with a major unmapped gene and a specific SLA (Swine Leukocyte Antigen) haplotype, noted as the “B haplotype,” or a modifying gene co-segregating with SLA. Later, Ho et al. (2010) have shown that the B haplotype actually corresponds to the SLA1 0201 and 0701 alleles. In MeLiM, a three-genes mode of inheritance was proposed by Hruban et al. (2004). More recently, different approaches were used to decipher melanoma occurrence, considering it as a complex trait, as detailed below for the MeLiM swine.

## Genetic Susceptibility in the MeLiM Model

Most of the genetic studies performed on the MeLiM model so far rely on an experimental backcross design. Briefly, four affected MeLiM were crossed to five healthy Duroc pigs, to produce an F1 generation. As described before, the Duroc breed presents a very small incidence of melanoma, and animals used were checked for the absence of cutaneous tumors. However, one cannot rule out the possibility that the Duroc pigs used previously harbored small tumors that underwent regression, or that without exhibiting lesions, they still carry predisposing variants that can be transmitted to the progeny. Sick F1 individuals were further backcrossed to Duroc animals to create a first backcross generation of 331 individuals (Geffrotin et al., 2004; Du et al., 2007). This three-generation pedigree was therefore used to test linkage and association of various genes with melanoma development.

Two genome-wide quantitative trait loci (QTL) analysis were performed after microsatellite genotyping of the MeLiM X Duroc cross (Geffrotin et al., 2004; Du et al., 2007). A first observation is that the inheritance of melanoma in this model is complex, likely autosomal dominant with an incomplete penetrance. Despite what is postulated in other models, linkage studies have shown that a 2 to 3-genes model is probably too simple to explain all the extent of the disease. However, the small incidence of melanoma in Duroc herds may add complexity to the results. The first linkage study, using only one MeLiM founder and its backcross progeny (*n* = 123), discovered 4 QTLs, on SSC1, SSC2, SSC7, and SSC8 (Geffrotin et al., 2004). The second QTL analysis used the extended backcross pedigree and focused also on more specific traits, such as clinical ulceration and invasion, or presence of metastasis for example. This linkage study led to the identification of various QTLs, some of which being detected for different phenotypes and thus potentially corresponding to genes with a pleiotropic effect (Du et al., 2007). All these studies were based on microsatellite genotyping. The availability of high-density SNP chips in pig should allow a more accurate detection of genomic regions associated to melanoma development and sub-phenotypes. An integration of genome-wide association results obtained in different melanoma-prone breeds would also be extremely powerful to detect major genes involved in melanoma predisposition in pigs.

On the other hand, a few candidate gene studies have targeted major actors of melanoma susceptibility in human. First, Le Chalony et al. (2003) used newly identified microsatellite markers in and around the *CDKN2A* locus to test the gene for involvement in melanoma susceptibility in the MeLiM pig. Association, linkage and haplotype analyses all excluded *CDKN2A* as predisposing in the progeny of one sick founder. However, the complex QTLs identified by the linkage analysis suggest the presence of one or several susceptibility gene(s) in the vicinity of the microsatellites tested. In humans, among cases linked to the HSA9p21 region (which encloses *CDKN2A*), half are not due to mutations in *CDKN2A*, thus suggesting the presence of another gene in the vicinity. A fine-mapping strategy could be worth setting up on the MeLiM model to try to evidence this other gene. In the first linkage study, Geffrotin et al. (2004) also excluded *CDK4* and *BRAF* as high risk genes, since microsatellites located near the loci did not segregate with melanoma. No linkage signal was observed in the second study for either gene (Du et al., 2007). The second QTL analysis highlighted a QTL on the swine chromosome 13, directly above *MITF* locus. However, subsequent studies showed no association between variants in the gene and melanoma phenotypes (Bourneuf et al., 2011). Also, the authors showed that the locus was not amplified in tumor samples, contrary to what is seen in humans. Yet, *MITF* expression seems tightly regulated during the course of the disease, showing as in humans, the probable central role of *MITF* in pig melanocyte biology.

A microsatellite located close to *MC1R* was linked to melanoma, even when the analysis was corrected for coat color, proving that pigmentation variation was not the only effect (Du et al., 2007). Two alleles were evidenced in the MeLiM swine: the allele *MC1R* E^D1^ in black animals and the E^p^ allele in red animals. These alleles correspond to the Asian black and the European black spotting alleles, respectively. Despite its name, a wide variety of colors exist for pigs homozygous for the latter allele (Fang et al., 2009). The *MC1R* E^D1^ allele, associated with melanoma in MeLiM pigs, carries a Leu102Pro polymorphism. This mutation is equivalent to the *sombre* mutation in mice, coding for a constitutive MC1R receptor (Robbins et al., 1993). Such a constitutive receptor induces a constant production of cAMP and steady activation of the subsequent signaling cascade. This would explain why a dark color is favorable to melanoma, which is opposite to what is seen in humans, where light phototypes have an increased risk. Interestingly, Tibetan pigs also carry the *MC1R* allele E^D1^ (Liu et al., 2016), but no skin lesion has been described in this breed to our knowledge. This confirms that *MC1R* is not sufficient to promote melanoma development but contributes to an increased penetrance.

In the first linkage study, a significant association was observed between melanoma development and a region on SSC8. The study evidenced that the Duroc alleles were promoting melanoma, when introduced on a MeLiM background. The *KIT* gene is located in this area, and is of utmost importance in pigment cell function as mentioned earlier. Also, *KIT* is mutated in different cancer types, including certain subtypes of melanoma. An association of a SNP in the exon 19 was shown, once again with the Duroc allele promoting melanoma. The exact mechanism explaining this association remains to be elucidated. An association between SLA microsatellites and melanoma was also observed. However, this result has not been confirmed by adding new individuals to the analysis. New methods for accurate genotyping of SLA (PCR-SSP) could be used to determine the specific haplotypes segregating in the MeLiM population and how they could influence melanoma development.

## Tumor Genetics in the MeLiM Model

So far, comparative genomic hybridization (CGH) was the only large-scale experiment performed to decipher somatic changes occurring during tumorigenesis in the MeLiM pig (Apiou et al., 2004). In this work, Apiou et al. (2004) laser-microdissected tumor cells, amplified DNA by DOP-PCR and compared it to genomic DNA extracted from pig lymphocytes. Some genomic gains were shared by NM and SSM subtypes of melanoma. However, only nodular melanoma showed a loss of material, located at 13q31-49. This loss was confirmed by I-FISH, which also indicated polyploidy of the tumors. This result is consistent with an hyperploidy that has been observed in cell lines from Sinclair melanoma. However, only a few gains and no loss were detected in both models, but experiments were performed on tissues in MeLiM, and cell lines for Sinclair.

## Future Directions

To go further in the comparison of the swine model with human melanoma, a more precise exploration of the tumor genome would be needed. A first question to address concerns the mutational burden in these tumors. Genome modifications should be limited since (i) regression systematically occurs, and thus may indicate that tumors do not escape immune surveillance using favorable mutations in specific genes (ii) the environmental UV-signature that is classically encountered in the genome of human cutaneous melanomas should be absent. Acral lentiginous and mucosal melanomas do not carry a genomic UV signature, but rather somatic signatures observed in other cancer types of unknown etiology, and are more subject to structural variants (Hayward et al., 2017). More specifically, the existence of recurrent mutations in oncogenes/tumor suppressor genes frequently found in human tumors should be investigated. In particular, a survey of coding mutations leading to neo-antigens would be a priority, since MeLiM pigs exhibit an efficient antitumor activity, notably through the humoral response. Preliminary studies in the model have shown the presence of anti-melanoma antibodies and the identification of targeted peptides is underway (Blanc et al., 2016). The TCGA network (The Cancer Genome Atlas Network) has now established a classification of cutaneous tumors according to their mutational status for genes BRAF, NRAS and NF1, all known as major actors of tumor initiation for melanoma. Tumors that do not fall into these categories are defined as “triple wild-type” and are more prone to focal amplifications or structural rearrangements (Cancer Genome Atlas Network, 2015). It is thus crucial to determine to which class the MeLiM tumors (and other swine melanomas) could be assigned. Also, the identification of somatic mutations in MeLiM that are similar to recurrent variation in human melanoma could pave the way to pharmacogenomics studies.

The recent CRISPR/Cas9 revolution has completely modified the landscape of genetic modification in many species, including pig. It is now feasible to introduce and target variation in the swine genome and produce quite efficiently modified animals, as shown in pilot studies (Lai et al., 2016). In the frame of the melanoma study, such a tool could have two attractive applications. First, genome modification could help validating a potential causal mutation for melanoma development. Second, CRISPR technology could help investigating the effect of a known mutation in human in a predisposed background and regressing model.

As mentioned previously, telomere function is currently a very active area of research in oncology. *TERT* (telomere reverse transcriptase) and some other components of the telomere maintenance complex have been identified as risk genes for several cancer types, including melanoma. In addition, *TERT* promoter is very frequently mutated in a wide range of tumors, enhancing *TERT* transcription, and thus favoring the reactivation of the telomerase system (Fredriksson et al., 2014). Among the 115 samples analyzed by the TCGA consortium to establish the genomic classification of cutaneous melanoma, 65% were found with an activating mutation in *TERT* promoter, and 7% with a focal amplification of TERT locus (Cancer Genome Atlas Network, 2015). In the Sinclair swine model, Pathak et al. (2000) evidenced a lack of telomerase activity and an increased number of abnormal karyotypic figures in cell lines derived from tumors. In MeLiM, no study has been conducted concerning telomere length and telomerase activity, however, Apiou et al. (2004) evidenced hyperploïdy in tumors. The absence of reactivation of the telomerase complex during oncogenesis in those pigs could be a factor participating to spontaneous regression. Also, one of the rare tumor types undergoing a spontaneous and complete regression in human, the neuroblastoma 4S, is associated with a range of factors, including an absence of telomerase activity (Brodeur and Bagatell, 2014). In conclusion, given that melanomas from the MeLiM model all regress spontaneously only a few months after the progression starts, it would be interesting to investigate the telomere length, and expression and functionality of the different components of the maintenance complex. A defect in the process could very well be a key to the regression phenomenon.

Finally, more advantage of the MeLiM model should be taken for better understanding the pathway alterations and molecular changes occurring between normal melanocytes and transformed melanoma cells. In addition, such a work could help evidencing new biomarkers to be tested in different species. Indeed, Egidy et al. (2008) explored the gene expression differences between a melanocyte cell line (PigMel) and a primary culture of melanoma lung metastasis from MeliM. This study pointed at *GNB2L1*, coding for RACK1, as a potentially interesting gene in melanoma characterization. Later, this scaffold protein with multiple functions has been described as a good marker for differentiating melanocytoma from melanoma in dog (Campagne et al., 2013) and horse (Campagne et al., 2012). More recently, Campagne et al. (2017) showed that RACK1 could cooperate with NRAS^Q61K^ mutation to accelerate melanoma onset and metastasis formation in transgenic mice. This example illustrates the usefulness of such a model to describe the role of pivotal molecules in specific pathologies.

## Study of Endogenous Retroviruses in Swine Melanoma

Recent reports have focused on human endogenous retroviruses (particularly of subtype HERV-K) and tumor biology. Endogenous retroviruses represent around 8% of the human genome, and correspond to an ancient integration of viral DNA in the germline genome. Several studies have shown a re-activation of HERV-K in various solid tumors, including melanoma (reviewed in Downey et al., 2015). HERV-K transcripts and proteins have been observed in melanoma cell lines and tissue (Buscher et al., 2006), but are absent from normal human melanocytes (Serafino et al., 2009). Current studies aim at better describing the regulation of expression of the different ERV transcripts, and their interaction with melanomagenesis. Notably, a correlation between HERV-K expression and the MEK-ERK and p16INK4A/CDK4 pathways was found (Li et al., 2010), and MITF-M was found to regulate the transcription of HERV-K LTR sequences (Katoh et al., 2011). More recently, Lemaitre et al. (2017) showed that HERV-K (HML-2) could even induce the ERK1/2 pathway *in vitro*, through upregulation of several transcription actors. The global hypomethylation of the genome observed in many tumors is likely to explain the reactivation of HERV transcription, usually silenced by CpG methylation of the LTRs (Stengel et al., 2010). Among the possible oncogenic mechanisms, HERV-K could participate to cell transformation by insertional mutagenesis, through its rec and np9 oncogenic proteins, or even by modulating the immune response (Gonzalez-Cao et al., 2016). However, even if HERVs are released as particles (which is rarely the case), they remain non-infectious (Denner, 2016). Nevertheless, many studies have identified a modulation of adaptive immune response mediated by ERV reactivation (Downey et al., 2015). In recent reports, the HERV-K env protein has been used as chimeric antigen receptor (CAR) expressed by T cells, therefore capable of inducing a promising anti-tumor response in murine models of melanoma (Krishnamurthy et al., 2015) or breast cancer (Zhou et al., 2015).

The existence of porcine endogenous retroviruses (PERVs) in the pig genome is one of the main barriers to pig-to-human xenotransplantation (Groenen et al., 2012). Indeed, even if most of PERVs are considered as defective, a risk of re-activation remains. For example, a higher transmission of PERV has been observed in mice xenografted with PERV-producing cells, in particular under an immunosuppressant treatment. Also, this PERV infection was correlated with a decrease of T cells proportion, especially CD4+ subset (Kim et al., 2016). In addition, SLC52A1 and SLC52A2 molecules are described as receptors for PERV-A particles on human cells *in vitro* (Colon-Moran et al., 2017), providing a demonstration of a possible infection of human cells by PERVs. In order to circumvent this infectivity, Yang et al. (2015) recently described the creation of a model of pigs where 62 PERVs have been knocked-out by the CRISPR-Cas9 technology. This work illustrates a renewed interest for heterologous transplantation, enabled by new methodologies. The existence and expression of PERVs were investigated in MMS swine (Dieckhoff et al., 2007). The authors found an enhanced expression of PERV transcripts in melanoma and metastasis compared to normal skin, as well as a release of viral particles in metastasis-derived cell cultures. Whether transcriptional activation of endogenous retroviruses in porcine melanoma is an initiator or a consequence of malignant transformation remains to be determined. Overall, swine models recapitulating tumor progression, an efficient immune response and a genome containing many active endogenous retroviruses could thus be of great help to decipher such intricate mechanisms.

## Conclusion

Swine models of melanoma exhibit several common features with human disease. Clinical and histopathological studies have shown a range of lesions that are comparable to different subtypes in human. Also, tumor heterogeneity has been observed at cellular and molecular levels. Genetically, pig melanoma is a complex trait with incomplete penetrance, and although high-risk genes remain to be discovered, *MC1R* has been involved beyond pigmentary phenotypes (**Table 3**). Efforts have to be pursued in order to fine-map already evidenced QTLs.

**Table 3:** Summary of findings on candidate gene studies in swine melanoma models.

Candidate gene	Model and approach	Results	Reference
**Cell cycle**			
*CDKN2A*	MeLiM; linkage, association and haplotype analysis	No linkage/association of the genes with melanoma occurrence	Le Chalony et al., 2003
*CDK4*	MeLiM, linkage analysis	No linkage with melanoma occurrence	Geffrotin et al., 2004; Du et al., 2007
**Pigmentation**			
*MC1R*	MeLiM; association analysis and sequencing	Association of *MC1R^∗^2* allele with melanoma occurrence	Du et al., 2007
*MITF*	MeLiM; linkage and association analysis, gene expression, somatic status	No linkage/association with melanoma occurrence; changes in gene expression compatible with human melanoma data; no genomic amplification in tumors	Bourneuf et al., 2011
*KIT*	MeLiM; association analysis	Association with melanoma occurrence	Fernández-Rodríguez et al., 2014
**Proliferation**			
*BRAF*	MeLiM; association analysis	No association with melanoma occurrence	Geffrotin et al., 2004
**Telomere biology**			
*TERT*	Sinclair; cytogenetics and functional assays	Karyotypic abnormalities, no telomerase activity in tumors	Pathak et al., 2000
**Endogenous retroviruses**	MMS Troll; cell biology	Increased expression of transcripts in melanoma and metastasis; release of viral particles	Dieckhoff et al., 2007


Of course, differences exist between the two species. One of the main discrepancies is the early age of onset of melanoma in pigs. While in human, most of tumors are caused by UV radiations and appear late in life, pigs are not exposed to sunlight and most tumors have a prenatal origin. The swine model can therefore help deciphering molecular mechanisms leading to melanocyte transformation independently of UV radiation. A survey of swine tumor genome variation may reveal recurrent mutations worth investigating in. Similarly, sampling and sequencing several lesions and metastasis from a same individual may illustrate precisely the heterogeneity and clonal origin of melanoma cells. Also, spontaneously developing and regressing porcine tumors could represent a valuable tool to study complex interactions between endogenous retroviruses, oncogenesis and adaptive immune response.

## Author Contributions

The author confirms being the sole contributor of this review and approved it for publication.

## Conflict of Interest Statement

The author declares that the research was conducted in the absence of any commercial or financial relationships that could be construed as a potential conflict of interest.
